# Comparison of liquid-liquid extraction-thin layer chromatography with solid-phase extraction-high-performance thin layer chromatography in detection of urinary morphine

**DOI:** 10.1016/S1674-8301(11)60048-1

**Published:** 2011-09

**Authors:** Ali Ahadi, Alireza Partoazar, Mohammad-Hassan Abedi-Khorasgani, Seyed Vahid Shetab-Boushehri

**Affiliations:** a Department of Physiology;; b Department of Pharmacology;; d Department of Medical Nanotechnology, School of Medicine, Tehran University of Medical Sciences, Tehran, I.R. 1449614535 Iran;; c Legal Medicine Organization of Iran, Tehran, I. R. 1449614535; Iran.

**Keywords:** morphine detection, liquid-liquid extraction, thin-layer chromatography, solid-phase extraction, high-performance thin layer chromatography

## Abstract

Liquid-liquid extraction-thin layer chromatography (LLE-TLC) has been a common and routine combined method for detection of drugs in biological materials. Solid-phase extraction (SPE) is gradually replacing the traditional LLE method. High performance thin layer chromatography (HPTLC) has several advantages over TLC. The present work studied the higher efficiency of a new SPE-HPTLC method over that of a routine LLE-TLC method, in extraction and detection of urinary morphine. Fifty-eight urine samples, primarily identified as morphine-positive samples by a strip test, were re-screened by LLE-TLC and SPE-HPTLC. The results of LLE-TLC and SPE-HPTLC were then compared with each other. The results showed that the SPE-HPTLC detected 74% of total samples as morphine-positive samples whereas the LLE-TLC detected 48% of the same samples. We further discussed the effect of codeine abuse on TLC analysis of urinary morphine. Regarding the importance of morphine detection in urine, the present combined SPE-HPTLC method is suggested as a replacement method for detection of urinary morphine by many reference laboratories.

## INTRODUCTION

Liquid-liquid extraction (LLE) has been a method of sample preparation for many years[1]–[4]. It commonly involves the direct extraction of the biological or non-biological material with a water-immiscible solvent. The isolation of the analyte is achieved by partitioning it between the organic and aqueous phases. An equilibrium distribution is established between the two phases, which follows the Nernst Distribution law. The distribution ratio between the two phases will be influenced by the choice of the extracting solvent, pH value of the aqueous phase and the ratio of the volumes of the organic to aqueous phases. The initial conditions of the extraction should be such that the analyte is preferentially distributed into the organic solvent. If there is a low recovery of the analyte, this can be enhanced by successive extractions of the sample to produce acceptable recoveries, but in practice it is often the case that a large excess of extracting solvent can be used in order to save time and achieve the same result[1]–[4].

Another method of isolating an analyte from biological and non-biological matrices is solid-phase extraction (SPE), which consists of adsorbent conditioning, passing the sample through the adsorbent (solid-phase)-containing column, adsorption of analyte by adsorbent, washing the interfering compounds, and eluting the analyte with an appropriate solvent. The success of this approach depends on the relative affinities of analyte between the sample matrix and the adsorbent and the relative ease of eluting the analyte for subsequent analysis. In other words, in this process, the sample passes over the stationary phase and the analytes are separated according to the degree of which each component is partitioned or adsorbed by the stationary phase. The analyte can be bound to the solid phase by a number of different mechanisms which are the same as for high-performance liquid chromatography (HPLC), i.e. hydrogen binding, dipole-dipole interactions, hydrophobic dispersion forces and electrostatic (ionic) interaction. The stationary phases (adsorbents) used in SPE are classified as nonpolar phases (e.g., C_2_-, C_8_-, and C_18_-bonded phase silica), polar and weak ion-exchange phases (e.g., silica, cyanopropyl-, and aminopropyl-bonded phase silica), and strong ion-exchange phases (e.g., propylsulphonic acid- and quaternary amino-bonded phase silica). SPE is much easier than LLE as it is considerably easier to separate a liquid from a solid than two immiscible liquids. The adsorbent provides the surface area necessary to ensure a high extraction recovery and cleanup. In general, SPE can be used for three important purposes in up-to-date analyses including concentrating of the analyte, removal of interfering substances, and changing the matrix of the analyte as needed for subsequent analyses[5]–[8]. SPE has several advantages over LLE such as low solvent consumption, enormous saving of time, increased extraction efficiency, decreased evaporation volumes, higher selectivity, cleaner extracts, greater reproducibility, avoidance of emulsion formation, and easier automation[5]–[8].

Thin layer chromatography (TLC) is one of the most useful, simple, inexpensive, rapid, relatively precise and sensitive methods for separation, identification, and quantification of drugs, poisons, and herbal medicines[9]–[14]. TLC and high performance TLC (HPTLC) have found many applications in analytical chemistry[15], analytical toxicology[16],[17], and pharmaceutical industries[18], including monitoring organic reactions and qualitative analysis of reaction products[19], detection of drugs and poisons in biological and non-biological materials[13],[16],[17], identifying compounds present in a given substance[9]-[14], assaying the purity of pharmaceuticals[18], and determination of the components of plant extracts[20],[21]. Alumina, kieselguhr, cellulose, and silica gel are among the adsorbents used as stationary phases in TLC[9]-[14]. Silica gel is by far the most widely used adsorbent in TLC[9]-[14]. TLC silica gel is a porous inorganic material, which is characterized by particle size (5-17 µm), pore size (60 Å), pore volume (0.75 ml/g), specific gravity (2.1 g/cm^3^), specific surface (BET) (500 m^2^/g), and pH stability (2-8)[9]-[13],[20]. HPTLC uses adsorbents of rather smaller sizes and hence results in increased sensitivity and decreased detection limit than that of TLC. Other advantages of HPTLC over TLC are smaller spot diameter before and after development, shorter migration distance and time, and decreased reproducibility of quantification and retention factor (R_f_) values[9]-[13],[20],[21]. For the standard silica plate used in TLC, the particle size is between 5 and 17 µm and the layer thickness is 0.25 mm for analytical plates[9]-[13],[20]. For HPTLC, 0.2 mm layers with a mesh size of 2-10 µm are applied[9]-[13],[20],[21].

In the present study, we sought to evaluate a new SPE-HPTLC method with a higher efficiency than the routine LLE-TLC method that is currently used in I.R. Iran Legal Medicine Organization at Tehran for the detection of urinary morphine.

## MATERIALS AND METHODS

### Chemicals and materials

Methanol, concentrated ammonia solution (25%), hydrochloric acid (25%), hexachloroplatonic (IV) acid, potassium iodide (all from Merck, Germany), morphine hydrochloride reference standard (Darou Pakhsh Pharmaceutical & Chemical Co., Tehran, Iran), pre-coated TLC polyester plates (silica gel 60 UV254, 20 cm×20 cm, 0.200 mm layer thickness) and pre-coated HPTLC aluminum plates (silica gel 60 UV254, 20 cm×20 cm, 0.200 mm layer thickness) (both from Merck, Germany), flat bottom TLC chamber for development of 20 cm×20 cm TLC and HPTLC plates (CAMAG, Switzerland), dual-wavelength (254/366 nm) UV cabinet (CAMAG, Switzerland), and LiChrolut® TSC SPE columns (300 mg, 3 mL, Merck KGaA, Darmstadt, Germany) were used in this study.

### Sample preparation by LLE and SPE

The study was performed on registered, coded, and labeled urine samples received by the toxicology laboratory of Legal Medicine Organization of Iran at Tehran. All human studies were approved by the ethics committee of Legal Medicine Organization of Iran and were performed in accordance with the ethical standards laid down in the 1964 Declaration of Helsinki. The samples were stored at +4°C until experimentation. Fifty-eight urine samples, primarily identified as morphine-positive samples by ACON® MOP One Step Opiate Test Strip (USA) according to the manufacturer's instructions, were selected and used in the present study. This test is a rapid chromatographic immunoassay which is on the basis of antigen-antibody immunochemistry for rapid qualitative screening of opiates in urine samples at a cut-off concentration of 300 ng/mL for morphine. Each 40 mL sample was divided to two equal portions of 20 mL. Each portion was examined by a separate laboratory personnel. The first portion was prepared for LLE followed by TLC and the second one was prepared for SPE followed by HPTLC. Both portions were treated as follows: To 20 mL-aliquot of each sample was added 1 mL of concentrated hydrochloric acid, which was then heated for 15 min at 100°C for breaking glucoronide conjugates. After cooling, the pH of the mixture was adjusted to 8-9 with concentrated ammonia. The first portion was extracted with 2×15 mL of chloroform-isopropanol (8:2). The organic phase was separated and evaporated to dryness under stream of nitrogen. Three mL of the second treated portion of each sample was used for SPE. A SPE system including a vacuum manifold for 12 columns (Macherey Nagel, Germany) connected to a vacuum pump (240 V, 50 Hz, 1/2 H.P., oil-less, 8 cfm, vacuum delivery of 30 in.Hg, Gast, USA, Model DOA-V130-BN) was used. SPE was performed on the second portion according to the manufacturer's instruction as follows: SPE columns were conditioned by addition of 2×3 mL of methanol, which was drawn slowly through the column at a flow rate not exceeding 2 mL/min. The vacuum was turned off as soon as the solvent reached the top of the sorbent bed to prevent column drying. Three mL samples were added to the columns and drawn slowly at a flow rate of 1 mL/min. Columns were cleaned from interfering components by passing 2×3 mL of distilled water through them at a flow rate of 2 mL/min. The columns were then dried under vacuum of 10 in.Hg to complete dryness. The analyte (morphine) was eluted from each column by passing 2 mL methanol:ammonia (9:1) through it without application of vacuum. Finally, the eluates were dried under the stream of nitrogen.

### TLC and HPTLC of extracted samples

Each LLE and SPE extract was dissolved in 100 µL of methanol and 5 µL of resulted solution was spotted on respective TLC and HPTLC plates by a micropipette. Moreover, 5 µL of a solution containing 60 µg/mL morphine reference standard was separately spotted on TLC and HPTLC plates to produce a reference morphine spot (300 ng) for comparison. The spotted plates were developed in a saturated TLC chamber containing ethyl acetate:methanol:ammonia (85:10:5)[22]. The plates were then dried under warm air. The morphine-positive samples were identified by visualization of morphine spots on TLC plates after uniform spraying[2] with acidified iodoplatinate reagent[23]. Rf and color of spots (violet) were two major parameters, which compared with that of morphine reference spot for identification of morphine-positive samples. Acidified iodoplatinate reagent was prepared as previously described[23]. The morphine-positive samples on HPTLC plates were identified by visualization of morphine spots under ultraviolet light at 254 nm in a UV cabinet.

### Statistical analysis

Statistical difference between LLE-TLC and SPE-HPTLC methods was determined by McNemar statistical test on the SPSS statistical package. A 2×2 contingency table was made and proportions of morphine-positive samples detected by SPE-HPTLC method were statistically compared with corresponding values of LLE-TLC method. The kappa index (κ) was calculated to measure strength of agreement between results of LLE-TLC and SPE-HPTLC methods. Differences were regarded as significant at *P* < 0.01.

## RESULTS

### The results of LLE-TLC and SPE-HPTLC methods

The R_f_ value of morphine, migration distance and migration time were 20, 15 cm and 35 min, respectively, for TLC method. The corresponding values for HPTLC method were 23, 10 cm and 25 min. Visual detection limits for TLC and HPTLC methods were 100 ng/spot and 300 ng/spot, respectively.

The results of LLE-TLC and SPE-HPTLC are summarized in ***Table 1***. They show that SPE-HPTLC significantly (McNemar's χ^2^ = 9.8, *P* < 0.001) detected more morphine-positive samples (about 25.86%) than that of LLE-TLC. The low kappa index (κ = 0.355) shows fair agreement between results of LLE-TLC and SPE-HPTLC methods. The results also indicate that SPE-HPTLC did not detect morphine in 25.86% (15/58) of the samples whereas LLE-TLC did not detect it in 51.72% (30/58) of the samples. Furthermore, the results indicate that both methods did not detect morphine in 22.41% (13/58) of the samples primarily identified as morphine-positive samples by ACON® MOP One Step Opiate Test Strip. Both methods also detected 44.82% (26/58) of the samples as morphine-positive samples.

**Table 1:** Comparison between the results obtained by LLE-TLC and SPE-HPTLC in 58 urine samples primarily identified as morphine-positive samples by ACON® MOP One Step Opiate Test Strip.

		LLE-TLC (*n*)		
		Positive	Negative	Total
SPE-HPTLC	Positive	26	17	43
	Negative	2	13	15
	Total	28	30	58

LLE-TLC: Liquid Liquid Extraction-Thin Layer Chromatography, SPE-HPTLC: Solid Phase Extraction-High Performance Thin Layer Chromatography. McNemar's χ^2^= 9.8, κ = 0.355, *P* = 0.001

## DISCUSSION

LLE-TLC has long been a combined method for screening of drugs of abuse[16],[24]–[26]. SPE has been most recently used for extraction of drugs and poisons with some advantages over LLE[2],[4]–[8]. The results show that SPE-HPTLC detected about 25.86% more morphine-positive samples than LLE-TLC. This may be due to the higher recovery rate of SPE than LLE. Higher recovery rate of SPE than LLE results in higher analyte concentration that reaches above the detection limit of HPTLC method.

The chromatographic results show that although the migration distance was longer for TLC, HPTLC had longer R_f_ value than that of TLC. This could be partly due to faster movement of mobile phase through wider macropores in TLC plates and partly due to entrapment of morphine in nanopores of larger-size TLC silica gel than that of smaller-size HPTLC silica gel[27],[28]. Furthermore, due to higher surface energy, smaller silica particles have stronger adsorptive properties than larger silica particles have[29].

One of the methods for reduction of detection limit in TLC is spraying the plates with suitable spray reagent i.e. derivatization[23],[30]. Although spraying the TLC plates with acidified iodoplatinate reagent reduces the detection limit of TLC method, it seems that higher extraction recovery of SPE than LLE causes the concentration of morphine to reach above the detection limit of HPTLC method in the present study. Although visualization of morphine spots on HPTLC plates under UV light at 254 nm has higher detection limit (300 ng/spot) than spraying with acidified iodoplatinate reagent (100 ng/spot), it has some advantages over the latter: it is a clean and inexpensive method which does not need expensive toxic spray reagents, spray equipments such as spray gun, spraying cabinet, and spraying pump with compressed propellant air or alternatively a rubber pump[9]–[11],[13],[16],[17],[31]. Furthermore, the HPTLC plate can be photographed under UV light at suitable wavelength (254 nm in this case) in a UV cabinet and kept for a long time as a digital photograph (digital documentation system)[9],[17],[32]–[34]. Because it is a non-destructive method, the analyte can be scraped off and kept for further analysis by other analytical methods[3],[14],[21]. Other non-destructive and reversible visualization method such as exposure to iodine vapors can be also used on the HPTLC plates[9]–[13],[17],[20],[26],[27]. Wavelength of applied UV light (254 nm) was not λ_max_ of morphine (due to instrument limitation) and is located on the right ridge of the first peak of morphine UV absorption spectrum. It is clear that by spraying HPTLC plates with visualization reagents (such as acidified iodoplatinate reagent), which are toxic, destructive, and expensive, the detection limit of this method for morphine will be further reduced. Furthermore, it is clear that by using a UV cabinet which can produce a UV light with λ_max_ of morphine, the detection limit of this method will be further reduced. However, our objective was to propose a safer, non-destructive, and inexpensive method which also has a higher sensitivity and a lower detection limit. SPE per se has higher recovery rate over LLE and so SPE-HPTLC has advantages over LLE-TLC.

The results also indicate that both methods did not detect morphine in 22.41% (13/58) of the samples, which were primarily identified as morphine-positive samples by ACON® MOP One Step Opiate Test Strip. It may be related to false-positive results of ACON® MOP One Step Opiate Test due to the presence of other drugs in the urine as the manufacturer has mentioned in the users' instruction manual. This assay provides only a preliminary analytical test result which should be confirmed by other analytical methods such as TLC or HPTLC. Furthermore, false-negative results due to detection of morphine-positive samples as morphine-negative samples by of ACON® MOP One Step Opiate Test have been reported[35]. A negative result may not necessarily indicate drug-free urine. Negative results can be obtained when morphine is present in sample but below the cut-off level of the test (300 ng/mL). LLE and SPE are sample pretreatment and preparation steps, which cause the analyte (morphine) to reach the detection limit of TLC and HPTLC and hence LLE-TLC and SPE-HPTLC have advantage over ACON® MOP One Step Opiate Test in detection of morphine. Adulterants, such as bleach and/or alum, in urine specimens and consumption of some drugs by abusers may produce negative results (false-negative) by ACON® MOP One Step Opiate Test[36]. LLE-TLC and SPE-HPTLC may seem to have time-consuming and laborious sample pretreatment and preparation steps (LLE for the former and SPE for the latter) but, due to their higher recovery rates, higher sensitivities and lower detection limits over ACON® MOP One Step Opiate Test, this disadvantage can be reasonably ignored.

In emergency conditions (for example hospital toxicology) and from legal standpoint, the presence of morphine in body fluids of abusers is a leading sign of morphine abuse. Practically, the quantitative determination of urinary morphine in many cases is unnecessary. Urine is the sample of choice for detection of many drugs because they are found at a concentration of about 100 times more than that of the blood[37].

Approximately 90% of a morphine dose is metabolized after administration[38]. The major metabolic reaction is conjugation with glucuronic acid to form morphine-3- and 6-glucuronides[38]. After an oral dose, 5-15 percent of codeine is excreted as free or conjugated morphine. Urine TLC analysis of users of codeine-containing pharmaceutical preparations will show 2 spots: one related to codeine and the other one related to morphine. Thus, laboratorians should be aware from the appearance of the morphine spot in these individuals. Urine TLC analysis of morphine abusers only shows one spot related to morphine. In users of codeine-containing pharmaceutical preparations, the intensity of the morphine spot will be about 5-15 percent of codeine spot due to the above-mentioned metabolism of codeine. Each country has its own laws regarding substance abuse, and the punishment for the detection of codeine and morphine in the urine will be different. In some countries, some OTC preparations contain codeine but in others, they are forbidden. In the former, it has been seen that some expert morphine abusers abuse codeine to deceive laboratorians who analyze their urine (unpublished results). In these cases, the laboratorians may suppose that the persons have used codeine-containing OTC preparations. They should be extremely careful in interpretating TLC analysis of these abusers. A leading sign for differentiation between morphine abusers and morphine abusers who abuse codeine to deceive legal authorities is that in TLC analysis of urine of the latter, the intensity of the morphine spot is 5-15 percent greater than that of codeine spot (unpublished results).

Although TLC and HPTLC have been previously used for separation, detection, and determination of opiates[17],[22],[24],[25], due to the aforementioned advantages of SPE over LLE and HPTLC over TLC, the current combined SPE-HPTLC method is suggested as an easy, rapid, sensitive, and new method for toxicology reference laboratories.
